# GOLIAH: A Gaming Platform for Home-Based Intervention in Autism – Principles and Design

**DOI:** 10.3389/fpsyt.2016.00070

**Published:** 2016-04-28

**Authors:** Valentina Bono, Antonio Narzisi, Anne-Lise Jouen, Elodie Tilmont, Stephane Hommel, Wasifa Jamal, Jean Xavier, Lucia Billeci, Koushik Maharatna, Mike Wald, Mohamed Chetouani, David Cohen, Filippo Muratori, MICHELANGELO Study Group

**Affiliations:** ^1^Electronics and Computer Science, University of Southampton, Southampton, UK; ^2^Department of Developmental Neuroscience, IRCCS Stella Maris Foundation, Pisa, Italy; ^3^Institute of Intelligent Systems and Robotics, University Pierre and Marie Curie, Paris, France; ^4^Department of Child and Adolescent Psychiatry, APHP, Groupe Hospitalier Pitié-Salpêtrière et University Pierre and Marie Curie, Paris, France

**Keywords:** autism spectrum disorder, Early Start Denver Model, serious game, intensive intervention, Imitation, Joint Attention, nomadic settings

## Abstract

Children with Autism need intensive intervention and this is challenging in terms of manpower, costs, and time. Advances in Information Communication Technology and computer gaming may help in this respect by creating a nomadically deployable closed-loop intervention system involving the child and active participation of parents and therapists. An automated serious gaming platform enabling intensive intervention in nomadic settings has been developed by mapping two pivotal skills in autism spectrum disorder: Imitation and Joint Attention (JA). Eleven games – seven Imitations and four JA – were derived from the Early Start Denver Model. The games involved application of visual and audio stimuli with multiple difficulty levels and a wide variety of tasks and actions pertaining to the Imitation and JA. The platform runs on mobile devices and allows the therapist to (1) characterize the child’s initial difficulties/strengths, ensuring tailored and adapted intervention by choosing appropriate games and (2) investigate and track the temporal evolution of the child’s progress through a set of automatically extracted quantitative performance metrics. The platform allows the therapist to change the game or its difficulty levels during the intervention depending on the child’s progress. Performance of the platform was assessed in a 3-month open trial with 10 children with autism (Trial ID: NCT02560415, Clinicaltrials.gov). The children and the parents participated in 80% of the sessions both at home (77.5%) and at the hospital (90%). All children went through all the games but, given the diversity of the games and the heterogeneity of children profiles and abilities, for a given game the number of sessions dedicated to the game varied and could be tailored through automatic scoring. Parents (*N* = 10) highlighted enhancement in the child’s concentration, flexibility, and self-esteem in 78, 89, and 44% of the cases, respectively, and 56% observed an enhanced parents–child relationship. This pilot study shows the feasibility of using the developed gaming platform for home-based intensive intervention. However, the overall capability of the platform in delivering intervention needs to be assessed in a bigger open trial.

## Introduction

Autism spectrum disorder (ASD) is a spectrum of neurodevelopmental disorders characterized by the presence of atypical social communicative interaction and behaviors (1). Typically, ASD is diagnosed through behavioral analysis in the 3–5 years age range and, once diagnosed, its treatment is mainly delivered through behavioral intervention following different intervention models. In essence, these models try to teach a child cognitive, social, and behavioral skills that are considered essential for independent living in the long run and various techniques have been developed over the years (2–7). However, two major problems associated with such interventions are as follows: (1) a person’s specific development intervention protocol, accounting for the actual difficulties and strengths of a child, needs to be designed to achieve maximal effects – ASD is a broad spectrum with significant inter-child variability, and it has already been established that tailor-made personalized intervention may be more effective compared to any generic type of intervention (8) and (2) at least 20 h/week are supposed to be needed for an intensive intervention (9, 10).

Characterization of a child is typically done through behavioral assessment by a trained therapist in clinical settings but such an approach is often prone to have subjective biases. To avoid such biases, one needs to employ a set of stimuli multiple times ensuring their repeatability and then extracting a set of objective measures for characterizing the outcomes. Repeatability is an essential criterion in this case so that an average performance measure in a stimulus-specific way could be obtained reflecting the child’s actual ability for responding to the stimuli in question. Such repeatability and the 20 h/week intensive intervention are difficult to achieve (10). In fact, its implementation needs a trained therapist and, given the prevalence of ASD, the workload of a therapist could make the effective implementation of this strategy impractical. Moreover, the involvement of trained parents/caregivers to be part of intervention also in home setting seems to be an effective strategy in order to increase the learning opportunity for children with ASD (11, 12). This requires parent training and regular monitoring to check whether the parents are implementing and properly adhering to the intervention protocol outlined by the therapist. However, the economic implication of such process is quite substantial.

In recent years, computer-based approaches have been shown to be effective in improving the learning cognitive and social skills of children with various learning disability conditions (13–15). In these methods, the target intervention is mapped into a set of computer games and is thereby training the children since children enjoy playing games rather than going through the conventional learning process (16–18). Most of these computer applications designed for people with autism focus on the relationship between one user and one computer and aim to help with specific behavioral problems associated with autism. Computers are motivating for children with autism due to their predictability and consistency, compared with the unpredictable nature of human responses. In regard to social interaction, the computer does not send confusing social messages. Research on the use of computers (19) for students with autism revealed increase in (1) focused attention, (2) overall attention span, (3) sitting behavior, (4) fine motor skills, (5) generalization skills (from computer to related non-computer activities); and decrease in (6) agitation, (7) self-stimulatory behaviors, and (8) perseverative responses. The importance of assistive technology for children with autism has been established by the fact that this technology can be used in rehabilitation for daily activities.

Motivated by these facts, we conceived a closed-loop system with computer gaming at its center that allows the interaction between subjects with autism and a partner. This approach may help in mitigating the effect of isolation that could affect the traditional computer applications mentioned above. The solution we developed is innovative because it seeks to go over the actual lacuna in various computer games for children with ASD. In fact, in most computer games for ASD, the children are engaged only with a computer screen. In our protocol children are engaged with another person (therapist/caregiver) who has a computer and share the activity with the child.

The intensity of intervention for ASD plays a crucial role in terms of clinical outcome. However, the hours of intervention assigned to children with ASD are usually less than the real need of the children. To mitigate this problem, the gaming platform is an interesting solution to increase (1) the hours of treatment for children with ASD and (2) involve caregivers in the intervention. The intensity of the treatment and the involvement of caregivers are two important requirements of the intervention in ASD. In this sense, the gaming platform is in line with the recent recommendation about the intervention proposed by Ref. (11, 12).

**Figure 1:**
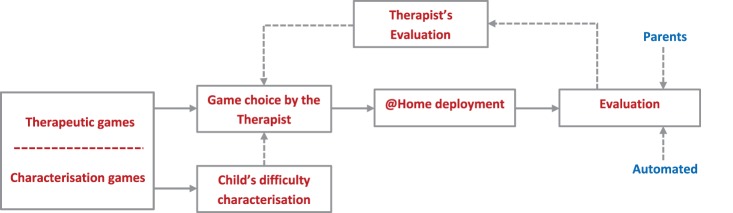
**The conceptual closed-loop intervention system**. The games contained in the platform are used for assessing the child, first, and for intervention purposes later. The first aim is to characterize the starting cognitive skills of a child by playing games at different levels of difficulty. After identifying the current level of ability of the child, a series of games and difficulties will be planned by the therapist and employed at home. According to the evaluation, both automated and manual, a new set of games will be planned by the therapist.

The conceptual view of a closed-loop system that may enable effective intervention integrating both the home and clinical settings is shown in Figure 1.

At the heart of the system is a computerized gaming library [Gaming Open Library Intervention for Autism at Home (GOLIAH)] that consists of a set of computer games created by mapping the desired intervention stimuli, Imitation, and Joint Attention (JA) in this case, into the games. In theory, the library could be divided into two parts – assessment games and intervention games – although they could be used interchangeably without loss of any generality. At the beginning, the child would be asked to play a set of games carefully selected from the library by the therapist for characterizing the child’s difficulties/strengths. Since a particular type of stimulus could be mapped in different ways in multiple games, this will allow using different games for ascertaining the child’s difficulties/strengths pertaining to a type of stimulus in a repeatable way without inflicting boredom on the child and thereby obtaining a much more precise average assessment of the child. Once characterized, the therapist could choose appropriate games (designated as the intervention games for convenience) from the gaming library that the child needs to play at his/her home setting on a regular basis adhering to a protocol outlined by the therapist. The aim here is to enhance the cognitive performance of the child through playing these games at home so that the effective intervention hours could be increased. The games could be made flexible enough so that the child may play the games with his/her parents (actively involving the parents without requiring an extensive training process) on a regular basis and with the therapist remotely connected through the internet at pre-scheduled times. The gaming system could have an automated evaluation process embedded in it that would extract a set of quantitative evaluation metrics, characterizing the child’s performance with each game and thereby providing the temporal evolution characteristics of the child’s performance. On the other hand, the parents could also assign a score manually according to a scoring criterion suggested by the therapist to signify how the child’s performance has evolved against each stimulus according to their own perception. All the automated and manually evaluated scores could be transmitted to the therapist who may compare them to check, on the one hand, how the child is improving and, on the other hand, whether the parents are adhering to the prescribed protocol truthfully. This could act as the basis of the evaluation by the therapist when he/she plays the game remotely with the child at a pre-scheduled time. Depending on this final evaluation, the therapist may choose a set of different intervention games from the gaming library once the child achieves the target set by the therapist and the whole process may continue. This closed-loop approach may help in alleviating several problems currently encountered by the autism therapists and have many advantages as described below in Table 1.

**Table 1:** **Advantages of the closed-loop GOLIAH approach**.

**Tailoring intervention through careful assessment of the child**
• Being computer based, the stimuli for assessment can be programed in an exact reproducible way
• The same type of stimulus could be mapped into different games giving the child the feeling that he/she plays different games. This is particularly important for assessing the child’s difficulties since repetition of the same game may force them not to respond to his/her capability level out of boredom. This fact is also true during the intervention stage
• Different difficulty levels could be incorporated within the games to ascertain the child’s performance even for a specific type of stimulus
• The whole process could be run automatically without incurring extra load on the therapist at the assessment phase
• A set of quantitative measures could be extracted in an automated way assessing the child objectively
**Nomadic intervention**
• The process could be deployed in nomadic environments where the child may play the game either with his/her parents or remotely with the therapist through internet connections
• Parents will need minimal training
• Automated measurements could give an objective idea about how the child’s performance changes over time in stimulus-specific way
• The therapist can adjust the intervention remotely and dynamically by adding/removing games from the pre-stored library
• It also opens up the possibility of a batch-mode intervention where the therapist may deliver intervention to multiple children located at various locations in one session

Improving social interaction skills of children with autism is a difficult task for their families as well as for well-trained therapists (20, 21). Although ASD remains a devastating disorder with a poor outcome in adult life (22, 23), there have been important improvements in the condition with the development of various therapeutic approaches. The literature on interventions in ASD has become quite extensive, with increasing convergence between behavioral and developmental methods (24, 25). The focus of many interventions is directed toward the development of skills that are considered to be “pivotal,” such as Imitation and JA (26–28).

Imitation plays a critical role in the development of every child. Among the several definitions of imitation, no definition is universally agreed upon: (1) Thorndike (26) offered a definition based on visual aspects: “learning to do an action by watching someone doing it.” However, a full definition of imitation must consider multi-sensory aspects. (2) Wallon (28) defined imitation as a learning technique without reward (or reinforcement). (3) Whiten and Ham (29) defined imitation as the process by which the imitator learns some behavioral characteristics of the model. Imitation fulfils two essential functions for adaptation: it is used for learning and it serves to communicate without words (30). Two children involved in imitation are temporally synchronized; they respond to the perception of movements or actions to produce a similar behavior. Compared to imitation, JA introduces a third partner during interaction. Emery defined JA as a triadic interaction that showed that both agents focus on a single object (31). Some authors (32) have argued that JA implies viewing the behavior of other agents as intentionally driven. In that sense, JA is much more than gaze following or simultaneous looking (33).

Lack of Imitation and JA are the main problems when interacting with children with ASD. While playing a game or conducting other activities with a social partner, these children tend to not concentrate on what others are actually doing, switching to repetitive and stereotypical behaviors that are of interest for the child but that usually have no or few relations with the actual social context. Imitation is possible but the communicative value of early imitation seems poorly understood (30). Also, children with ASD can display concerted attention to toys or objects that they like, but they have difficulties in sharing attention or interests with others (34). For example, maintaining eye contact with the caregiver is especially complicated (35, 36) and the lack of JA is the consequence (37, 38).

Owing to the importance of Imitation and JA as core difficulties in ASD, we mapped a subset of related stimuli from the Early Start Denver Model (ESDM) protocol into the gaming platform containing a set of games with varying levels of difficulties that could be dynamically adjusted by the therapists. This program aims to meet the socio-emotional needs of children and their families, to identify and use validated and effective intervention techniques that are based on developmental needs (39). The ESDM recently received strong evidence of its efficacy at the level of clinical outcome (40) and brain plasticity (2).

Motivated by these facts, the purpose of the work is to design a novel computerized gaming platform that would allow: (1) delivering intensive intervention in nomadic environments for Imitation and JA tasks in children with autism, (2) tailoring and adapting intervention through child-specific assessment of difficulties, (3) enhancing effective intervention hours, and (4) without increasing the cost of delivery. The major point to note here is that GOLIAH is not intended to replace one of the state-of-the-art interventions for ASD but to supplement and expand it for achieving its maximal benefit.

## Methods

### Participants

We tested the software in a 3-month open trial with 10 children with ASD (all boys, aged 5–9 years) to assess the performance of the software itself. All children were recruited in the Department of Child and Adolescent Psychiatry, Hôpital Pitié-Salpêtrière, Paris and in the Department of Child Neuropsychiatry, IRCCS Stella Maris Foundation, Calambrone, Pisa. The study was approved by the local ethics committees of each institution (*Comité de Protection des Personnes* Ile De France VI under agreement number CCP 21-14, and Comitato Etico of the Stella Maris under agreement number 05/2011) and was in accordance with the declaration of Helsinki. Each parent gave informed written consent before inclusion for participation and for publication of the individual clinical data. Clinical characteristics of the children are given in Table 2.

**Table 2:** **Socio-demographic and clinical characteristics of the participants**.

	ASD (*N* **=** 10)
Age, mean (±SD)	6.8 (±1.4)
Male – female	10 – 0
**ADI-R, current, mean (±SD)**	
Social impairment score	14.14 (±4.58)
Communication score	10 (±5.82)
Repetitive interest score	4 (±2.91)
**Cognitive Level (WISC3/WPPSI)**	
VIQ	103.1 (±14)
PIQ	96.1 (±24.8)
**Vineland: mean (±SD)**	
Communication score	88.2 (±16.7)
Daily living score	84.3 (±13.4)
Socialization	79.5 (±10.3)

*ASD: Autistic Spectrum Disorder; ADI-R: Autism Diagnostic Interview-Revised; WISC 3: Wechsler Intelligence Scale for Children 3; WPPSI: Wechsler Preschool and Primary Scale of Intelligence; VIQ: Verbal Intelligent Quotient; PIQ: Performance Intelligent Quotient*.

### Procedures

The intervention protocol used with children included six sessions per week (from Monday to Friday) of training with GOLIAH; five sessions per week were at home with the parents (mother or father) playing with their children in the afternoon; and one session per week was planned at the hospital. The duration of each session, both at home and at hospital, was equal to 20 min. The sessions at home and at hospital were the same in terms of tasks. The only differences were the different setting (i.e., home or hospital) and the partner (therapist or parent). Each child’s plan was tailored on the basis of functional profile and adapted during the 3-months protocol according to children progress in playing the games. This open-trial aimed at assessing (1) the usefulness of the gaming platform with children–therapist interactions as well as with children–parents, (2) whether tailored intervention was useful when used at home and with non-professional therapist/parents, and (3) whether children performed as expected when using the different Imitation and JA games. To do so, we used both objective data computed from the platform and clinical annotations produced by therapists during weekly sessions at hospital. (4) Finally, subjective views from users were also explored through a questionnaire.

At the beginning of the study, a 3-month open trial was planned with 60 sessions (four sessions at home per week + one session at the hospital per week = five sessions per week × 12 weeks = 60 sessions). To assess in detail the usability of the gaming platform, we planned a systematic recording of the number of times each game was played in each session by each of the 10 children included in the 3-month study period. Details are shown individually in Table 3.

**Table 3:** **Number of sessions per game and per child during the 3-month study period**.

Child	1	2	3	4	5	6	7	8	9	10	N of sessions per game for all children: mean (range)
**Imitation games**											
Imitate free drawing	11	4	4	6	3	19	16	19	15	16	11 (3–19)
Imitate step by step draw	17	13	24	10	5	20	11	18	13	9	14 (5–24)
Imitate speech	17	13	15	9	11	15	11	19	12	6	13 (6–19)
Imitate sounds	2	19	10	13	11	10	17	9	11	8	11 (2–19)
Imitate actions	15	23	7	6	10	14	11	14	4	16	12 (4–23)
Imitate actions and build	12	11	19	13	12	12	14	11	12	13	13 (11–19)
Guess the instrument	4	3	11	10	9	2	1	7	6	5	6 (1–11)
**Joint attention games**											
Follow the therapist’s pointing	15	19	20	17	12	14	13	16	21	12	16 (12–21)
Cooperative drawing	2	19	15	11	13	9	11	11	18	18	13 (2–19)
Bake a cake	11	14	16	15	12	18	9	12	19	7	13 (7–19)
Receptive communication	21	25	31	20	17	16	15	25	9	12	19 (9–31)
**No. of sessions per child for all games: mean (range)**	12 (2–21)	15 (3–25)	16 (4–31)	12 (6–20)	10 (3–17)	14 (2–20)	12 (1–17)	15 (7–25)	13 (4–21)	11 (5–18)	

### Instruments

#### Software Design

The game software has been developed in Microsoft Visual Studio 10 Platform in C# language. The platform has as many classes as the number of included mini-games; thus, creation of new games will not alter the existing ones. Real-time communication between two devices is performed through a multi-threading process that includes: (1) game flow thread in which all the game tasks are performed (including sending objects to the other user) and (2) receiving thread in which the objects sent by the other user are received and fire the semaphore in the game flow thread. The two players are connected to a server, developed in C#, which acts as a bridge between them. In fact, the objects exchange occurs through a Socket connection based on a TCP/IP protocol that ensures that the information exchange will not be lost during the transmission.

#### Choice of Stimuli

The ESDM is a comprehensive behavioral early intervention protocol for children with autism. It uses a combination of developmental and behavioral techniques in both therapist and parent-implemented early intervention models (41, 42). It is an intervention for infants with ASD aged 12–48 months that combines applied behavior analysis (ABA) with developmental and relationship-based approaches. The intervention is provided by trained therapists (Antonio Narzisi is a certified therapist from MIND Institute, University of California Davis, Davis, CA, USA) and parents.

Each child’s treatment program includes models based on development, functional profile, relational patterns, and modification of behaviors. The curriculum includes, among others, systematic activities on receptive and expressive communication, as well as social, play, cognitive, self-care, and fine and gross motor skills. Particular attention is devoted to specific tasks regarding Imitation and JA. ESDM considers JA as an activity in which two subjects are engaged with each other in the same cooperative activity, attending to the same objects, or playing or working together on a common activity. A JA routine is made up of several phases: (1) the opening or set-up phase that involves the acts that precede the establishment of the first shared play activity based on the theme of the play. (2) The child and adult are engaged in a definable play activity, either object centered, such as building blocks, pouring water, marking with crayons, or involving a social game, such as singing a song, dancing to music, or playing hide and seek. (3) The elaboration phase involves variation on the theme to keep it interesting or to highlight different aspects of the activity. This preserves the play from becoming repetitious and allows more skill areas to be addressed. (4) The closing is the fourth and final phase when attention is waning or the teaching value of the activity is all used up. It is a time to put materials away and to transit to something else. Closing allows nice transitions in changing activity, location, and time.

Regarding imitation, in the ESDM different tasks may be proposed to the children: (a) imitation of actions on objects, (b) imitation of gestures, and (c) vocal imitation of sounds and words. During intervention sessions, children are asked to imitate conventional or unconventional actions with and/or without objects using or not the vocalizations.

#### Mapping ESDM Stimuli for Imitation and JA into a Computerized Gaming Platform

The Imitation and JA stimuli are mapped into 11 games: seven Imitation and four JA games. Although currently the proposed platform consists of 11 games, it is flexible enough for developing/adding new games according to the need. A list of the games and the ESDM stimuli they address is depicted in Table 4. In developing the games, special attention has been devoted to their realistic resemblance to the real-life scenario, more importantly emulating human–human interactions during the game playing phase. Each of the games incorporates different levels of difficulty ranging from the application of one stimulus (e.g., the sound of a train), to a combination of different stimuli (e.g., the sound and the image of a train).

**Table 4:** **Mapping of ESDM stimuli for JA and imitation into the games**.

Game type	Description	ESDM stimuli
Imitation game 1: imitate free drawing	Imitation of the drawing done by the online therapist/parent	(lev.4) FM 4
Imitation game 2: imitate step by step drawing	Imitation of a drawing created step by step from the online therapist/parent (three difficulties)	(lev.4) FM 4
Imitation game 3: imitate speech	Imitation of words or phrases from the library (three difficulties)	(lev.2) IM 3, 9
Imitation game 4: imitate sounds	Imitation of sounds chosen from the library (four difficulties and two categories of stimuli)	(lev.2) IM 2
Imitation game 5: imitate actions	Imitation of the actions with balls made by the online therapist/parent (three difficulties and two types of task)	(lev.2) IM 6
Imitation game 6: imitate actions and build	Imitation of the actions with cubes made by the online therapist/parent (three difficulties and two types of task)	(lev.3) FM 3
Imitation game 7: guess the instrument	Identification of the musical instruments played and chosen by the therapist/parent from the library (two difficulties)	(lev. 1, 2) IM
Joint attention game 1: follow the therapist’s pointing (both audio and visual)	Identification of the object indicated (verbally, visually or pointed) by the therapist on the video and chosen from the library (six difficulties and eight categories of stimuli)	(lev.1) RC 1, 4 (lev.2) JA 2, 4, 6
Joint attention game 2: cooperative drawing – connect dots	The therapist and the child cooperate to complete a figure shown on the right, by clicking on the corners of the figure itself (two difficulties and four categories of stimuli)	JA
Joint attention game 3: bake a cake	The child cooks a recipe by clicking and dragging into a bowl the ingredients chosen by the therapist/parent from the library of recipes (11 categories of stimuli)	JA
Joint attention game 4: receptive communication	The child identifies the objects described by the therapist/parent and chosen from the library (three difficulties and five categories of stimuli)	(lev.2) RC 5, (lev.1) RC 6, (lev.1) RC 4

*FM, fine motor subset; IM, imitation subset; RC, receptive communication subset; JA, Joint Attention subset*.

The seven Imitation-based games comprise of tasks involving the imitation of drawing, speech, sounds, and building actions. For instance, the one related to the sound imitation (Imitation game 4) requires the child to repeat the sound played on the device, either a tablet or a computer. Whereas in the building action game (Imitation game 6), the child would build an object, starting from simple cubes, in a similar way to a normal session with Lego toys. The other four games are based on JA stimuli, including the identification of objects (such as fruits, home furniture, and vehicles), described or pointed to by the therapist/parent.

#### The Gaming Platform

The multi-player gaming platform developed here requires two computers or tablets with an active internet connection. One computer/tablet is operated by the therapist or parent (depending upon the application scenario) acting as the *therapist/parent* and the other by the child designated as the *player*. Currently, the platform is available in three different languages (Italian, English, and French) for providing instructions to the child and the therapist/parent.

The choice of the language, the game to play as well as the goal setting is made by the therapist/parent. As instance, when playing the musical instrument game, the *therapist/parent* can select between two different goal settings: listen and recognize a sequence of (a) three or (b) six musical instruments. The role of the player is to achieve the goal set by the therapist/parent at the end of the game. In the game described above, the child will listen to a sequence of instruments and, depending on the goal selected, he will listen and recognize the sequence of three or six instruments.

The games can also be categorized in (a) stand-alone operation game and (b) game requiring active co-operation between the therapist and the child. (a) The stand-alone operation games contain pre-developed libraries containing the stimuli and the instruction to achieve the goal. The imitation game 4 – Imitate Sound is an example of stand-alone game; the therapist/parent selects a list of animal’s sounds to imitate: the player will listen to each sound and imitate it. (b) In the second category of games, the therapist/parent has an active role: he/she needs to cooperate with the child to achieve the goal of the game and can also create new stimuli. An example of this category is the JA game 2 – Cooperative drawing-connect dots: both therapist/parent and the child have to cooperate to connect the dots and create the final figure. Details and figures of these games can be found in the supplementary material.

All the games have different levels of difficulty allowing the therapist/parent to adjust the initial level of difficulty according to the cognitive skills identified by the therapist at the beginning of the treatment process or dynamically adjusting it as the player’s performance progresses with time.

The performance of the player could be assessed mainly in two different ways: through an (a) automated evaluation based on a predefined scoring convention and through a (b) manual evaluation by the therapist/parent. (a) The automated evaluation does not require any action to the therapist/parent: the game will automatically assign a score to the performance of the child. For example, the game will assign a positive score if the child has selected the right musical instruments. (b) The manual evaluation requires to the therapist/parent to select among three different buttons: score 0 if the player did not achieve the goal, 1 for partial achievement, and 2 for successfully satisfying the goal. As instance, at the end of the imitation game 4 – Imitate Sound, the therapist/parent has to click among three buttons indicating score 0, 1, or 2. Without loss of generality, a more complicated scoring system could be programed easily according to the need of granularity to assess the achievement of the player.

Apart from the simple scores describing whether the player has achieved the goal, a set of objective metrics and an array of possible events are also extracted by the platform in an automated way. A list of such objective measurements is given in Table 5 along with their definitions.

**Table 5:** **The objective metrics extracted by the gaming platform**.

Measurement type	Measured metrics	Description
*Automated*	Name of stimulus	Type of the stimulus embedded within the game and the name of the object the player has to click or drag or draw
	Time of the stimulus	Defined by the difference ΔT_s_ = T_ss_–T_es_ between a start time T_ss_ variable (the time instant the stimulus starts to be shown or played on the child’s device) and an end time T_es_ variable (the time instant the stimulus is finished)
	Time of response	Defined by the difference ΔT_r_ = T_sr_–T_er_ between a start time T_sr_ variable (the time instant the child starts to respond) and an end time T_er_ variable (the time instant the child complete his/her response)
	Type of response	Defined by the correctness of the child’s response depending on whether the child performs action as intended by the therapist/parent (only Correct or Incorrect)
	Score of response	Assigned score to the response of the child, either 1 or 0 signifying whether the intended response has been achieved or not respectively – a more complicated scoring system could be programed
	Image of the stimulus and the response	A screenshot of the child’s device obtained during imitation drawing and the action games – assisting the therapist to analyze the response further offline to ascertain the quality of response.
	Sound recording	The audio response of the player recorded during the sound and speech imitation games – allowing the therapist to check the quality of response
*Manual*	Therapist/parent evaluation	Defined as Complete/Partially complete/Incomplete response of the child according to the therapist/parent judgment
	Manual score	Assigned to 0/1/2 corresponding to the therapist/parent evaluation of the child’s action – a more complicated scoring system could be programed

This set of objective metrics allows the therapist to analyze quantitatively the performance of the player in a stimulus-specific way not only at a particular time point but also the progression of the child’s performance over a time window (hours, days, months, etc.) giving a holistic picture of the child’s development. For example, the therapist might want to analyze if the child recognize a particular musical instruments and if this recognition becomes quicker throughout the sessions. In addition, the objective metrics allow the therapist to ascertain the appropriateness of scoring and adherence to the prescribed protocol by the parents. Such analysis could be done both online and offline by the therapist as the metrics are stored each time the player plays the game. For example, in the imitation game 1 – Free drawing, both the therapist/parent and the player’s drawing are saved as well as the scores given by the parent. The therapist could then check if the parent’s scores adhere to the scoring guidelines suggested by the therapist.

The gaming platform provides a flexible means for giving a reward to the player on successful completion of the goal capturing the essence of reward-based intervention. In the current version, a smiley face is shown at the end of each game in the player’s device, regardless of the score obtained as a positive reinforcement that also gives an impression of feedback to the player. Such feedback is once again programmable and an appropriate reward could be set by the therapist depending on the player’s motivation factors (such as playing music that the child likes, etc.).

### Descriptions of the Games

**Figure 2:**
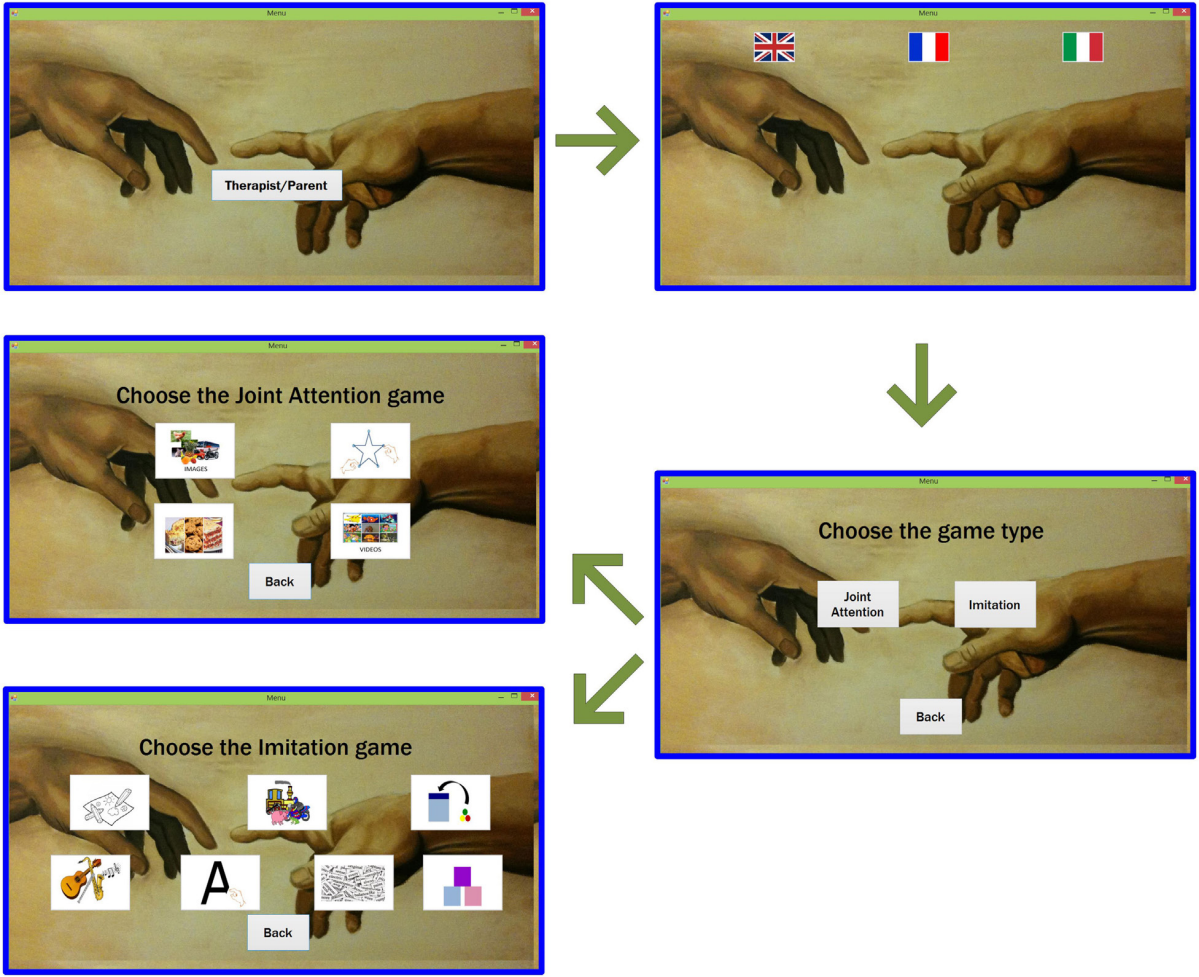
**Main windows of the therapist/parent during the beginning of the game**. The therapist/parent (blue windows) will select the language, the category of the game (whether Joint Attention or Imitation), and the game, according to the category chosen.

At the start of the game, the main window, shown in Figure 2 will appear on the therapist/parent’s device. He/she will first choose the language in which the stimuli and instructions will be played. Thereafter, the therapist/parent selects the desired game that will automatically be launched on both devices.

Here, we report only the description of two games (Free drawing and Bake a recipe) and we use it to illustrate the children’s performances through sessions of both Imitation and JA (a detailed description of all other games is reported on Supplementary Materials GamesDescription.doc).

#### Joint Attention Game 3 – Bake a Recipe

**Figure 3:**
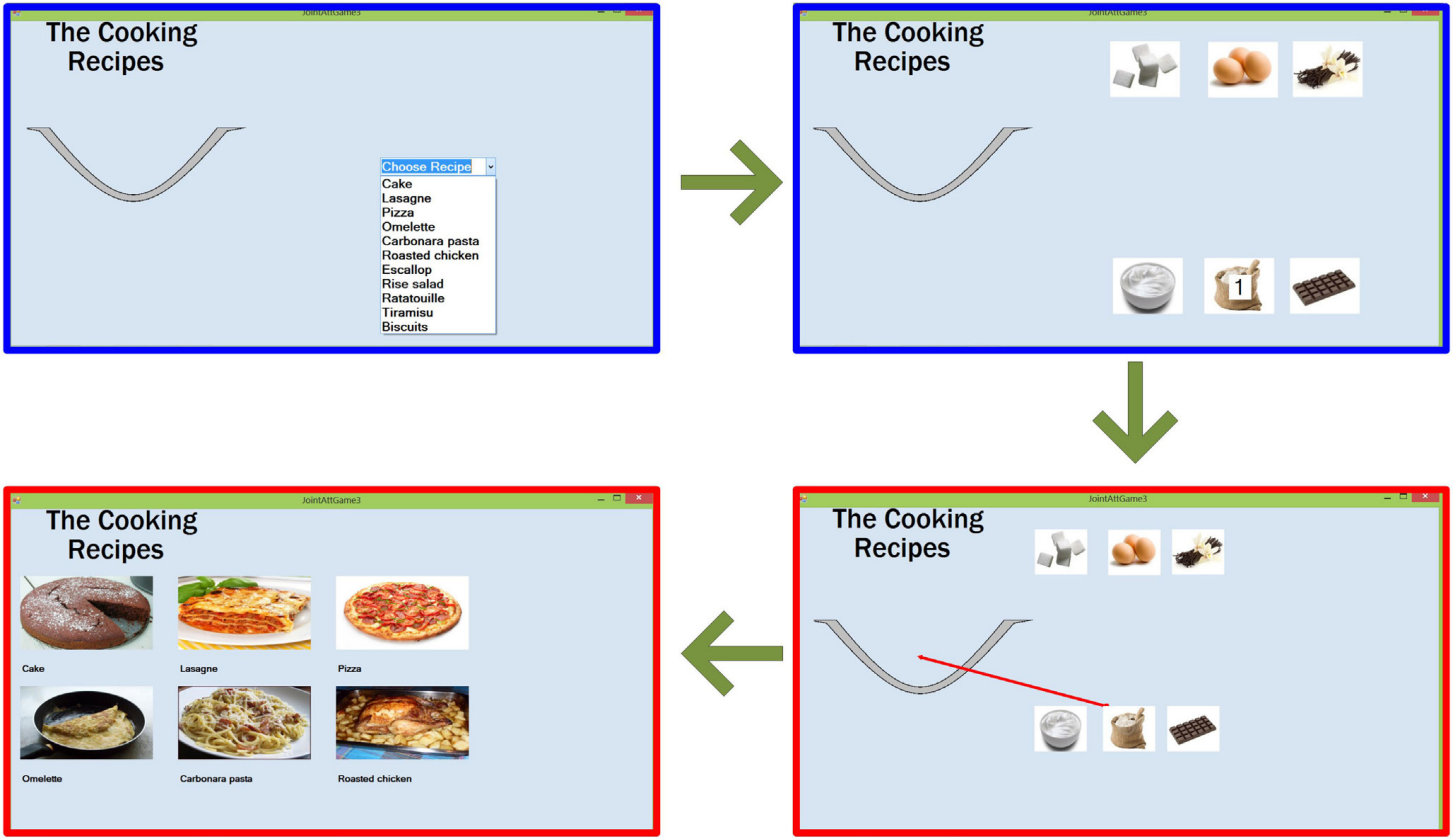
**Flow of the Joint Attention game 3 – Bake a recipe**. The therapist/parent (blue windows), after selecting the recipe, will select each ingredient to be dragged into the bowl. The red arrow on the player’s device (red window) will indicate the ingredient selected by the therapist/parent. After dragging all the ingredients, the player’s will click on the recipe cooked.

This game is targeted to cook a recipe by mixing six ingredients in a bowl, as shown in Figure 3. The therapist/parent selects the recipe to cook among 11 dishes from a standardized library, which includes pizza, tiramisu’, lasagne, omelet, roasted chicken, pasta, etc. For each of the six ingredients, as soon as the therapist/parent clicks on it, an arrow connecting this ingredient to the bowl appears on the player’s device, as shown in Figure 3. The player needs to drag the ingredients into the bowl. When all the ingredients have been dragged into the bowl, the player has to click on the Mix button and, finally, he/she has to choose the recipe they cooked among seven dishes.

As before, an event with positive or negative score is generated each time the player clicks on an ingredient and drags it into the bowl, as well as when the correct recipe is recognized.

#### Imitation Game 1 – Free Drawing

**Figure 4:**
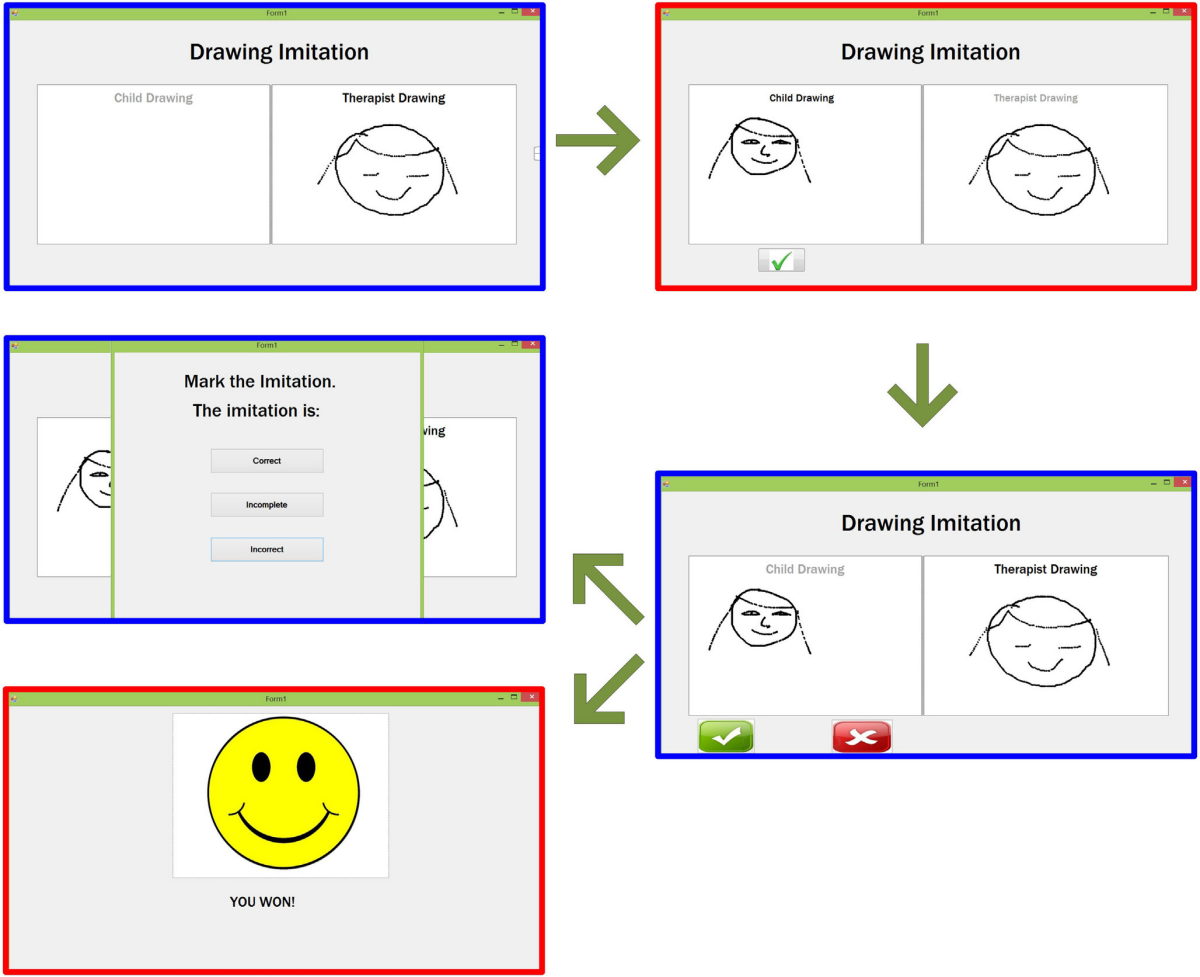
**Flow of the Imitation game 1 – Free drawing**. The therapist/parent’s drawing (blue window) appears on the player’s window (red window) who will then imitate the drawing and send it to the therapist/parent. After the therapist/parent’s feedback, the smiley will appear on the player’s device, while the therapist/parent will evaluate the imitation as Correct/Incomplete/Incorrect.

This imitation game is intended for examining the player’s ability to imitate several objects drawn by the therapist/parent, starting from very basic drawings, such as scribbles and dots, to very complicated, such as letters and numbers. The whole process of this game is shown in Figure 4, where the blue window indicates the therapist/parent’s window and the red window indicates the player’s window. Once launched, a window will appear on both therapist/parent and player’s device with clearly marked separate drawing panels. The therapist/parent can draw any object of any shape in the panel dedicated to him/her (on the right). Once completed, the therapist/parent’s drawing appears on the player’s device and the player needs to imitate that drawing in his/her dedicated panel (on the left). The live outline of the player’s drawing will appear on the therapist/parent’s device. Depending on whether the drawing is correct or not, the therapist/parent can decide to finish the game (by clicking on the tick button) or encourage the player to have another try (by clicking on the cross button). The quality of the imitation will be evaluated by the therapist/parent among three possibilities: correct, incorrect, or partially correct. To avoid discrepancies and to create normalization, the therapists involved in this study have reached an agreement, according to the ESDM, on how to evaluate the drawings and sounds imitation and train the parents to adhere to it.

## Results and Discussion

### Validation by Testing with Children

Overall, during the study period, the children and the parents participated in 77.5% of the planned sessions at home and in 90% of the hospital sessions. All children went through all games (seven Imitation games and four JA games). Given the diversity of the games and the heterogeneity of children profile and abilities, for a given game the number of sessions dedicated to the game varied. Also given the levels of difficulty within a game, all the conditions of the games have not been exploited by the children at the end of the 3 months. All games were well tolerated and followed both by children and parents showing the robustness of the gaming platform and the feasibility of the course of the games. One family initially had troubles in using the two tablets system related to Wi-Fi connecting problems that could be easily corrected. Tailoring treatment during the hospital session and data transfer from home was easily achieved.

### Children’s Performance through Sessions and Games

We selected two games to illustrate the children’s performances through sessions of both Imitation and JA by using either quantitative or qualitative scoring. Our goal here was to verify how meaningful the extracted scores were from each game session to follow the child’s progress or difficulties.

#### Bake a Recipe (Joint Attention Game 3 – Quantitative Scoring)

**Figure 5:**
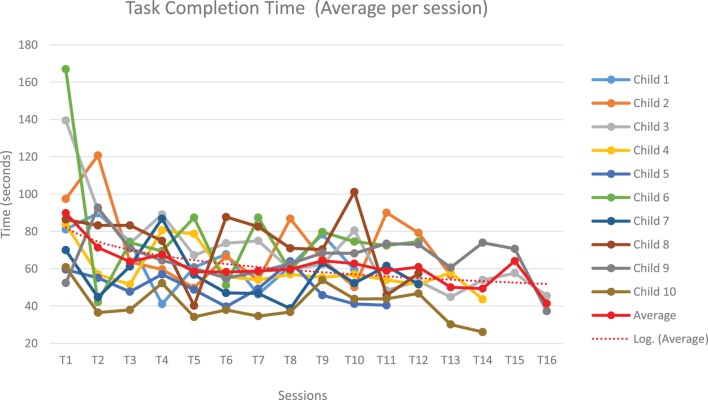
**Evolution of the time (in seconds) to complete the task for the Joint Attention game 3 – Bake a recipe**. Evolution of the time occurred to complete the Joint Attention game 3 for each child (each color represents a child) across different sessions. The average across children, in dotted red, shows a decreasing trend across sessions.

**Figure 6:**
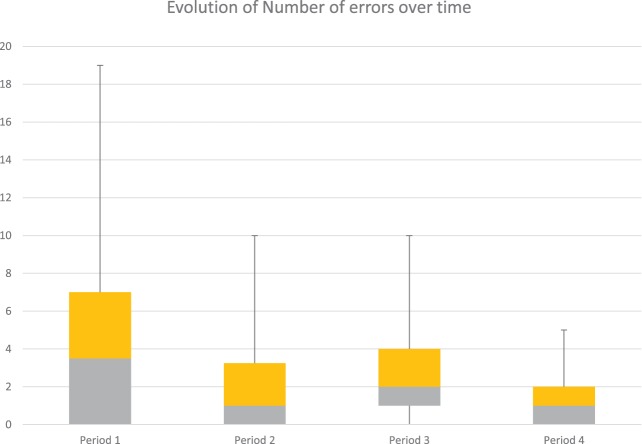
**Number of errors performed to complete the task for the Joint Attention game 3 during different periods**. The figure contains the number of mistakes committed by the 10 children during the Joint Attention game 3. The total number of errors decreases across the different periods, as shown by the variability (from 19 to 5).

Figures 5 and 6 show children’s performances for the JA game 3 – Bake a recipe. Figure 5 represents the evolution of the time (in seconds) to complete the task for the JA game 3. For one session (T_i_, T_i+1_ …), completion time is averaged, as the children practice the game several times during one session. As sessions progressed over time, children become faster to achieve the task. Each line corresponds to the evolution of the task completion time across different sessions for a given child. The red dot curve represents the evolution of task completion time averaged for all children (*N* = 10): a common overall decrease was observed in all subjects. To assess whether the task completion time significantly decreased over the sessions, we used a linear mixed model with the Log (time to complete the task) to be explained by the number of sessions as a continuous variable. The Log function was required to have a normal distribution. We found that the time to complete the task significantly decreased along sessions (β = −0.021, *t*-value = −5.53, *p* < 0.001).

In parallel, the number of errors decreased also over time (Figure 6). For this game, the mistakes that have been taken into account are as follows: wrong and fake answers during the first “mixing ingredients” phase of the game (when the child selects the wrong ingredient or when he presses one or several wrong ingredients after selecting the correct one) and wrong answers during the “choose recipe” phase of the game (when he/she has to guess the cooked recipe). For reasons of readability of the boxplot type graph (Figure 6), the sessions have been grouped into four periods (period 1 = T_1_, T_2_, T_3_, T_4_; period 2 = T_5_, T_6_, T_7_, T_8_; period 3 = T_9_, T_10_, T_11_, T_12_; and period 4 = T_13_, T_14_, T_15_, T_16_).

According to our data, the children who had already good performances at the beginning (Period 1), kept their performances constant all along. But there is an important decrease of the number of errors per child across the four periods, particularly for the children who committed several mistakes initially. At the end (Period 4), the number of mistakes is very low for all children. To assess whether the number of errors significantly decreased over the number of sessions, we used a linear mixed model with a binomial variable (the probability of correct answers) to be explained by the number of sessions as a continuous variable. We found that the probability of correct answers significantly increased with the number of sessions (β = 0.039, *z*-value = 2.78, *p* = 0.005). In sum, for this game, the results after 3-month training are promising.

#### Free Drawing (Imitation Game 1 – Qualitative Scoring)

For the second game (Imitation game 1 – Free drawing), the evolution of performances is illustrated from the results of one child, since the results are mainly qualitative and it is difficult to compare the drawing performances of one child with another (complexity of pictures, differences in drawing time, differences in fine motor skills, etc.).

**Figure 7:**
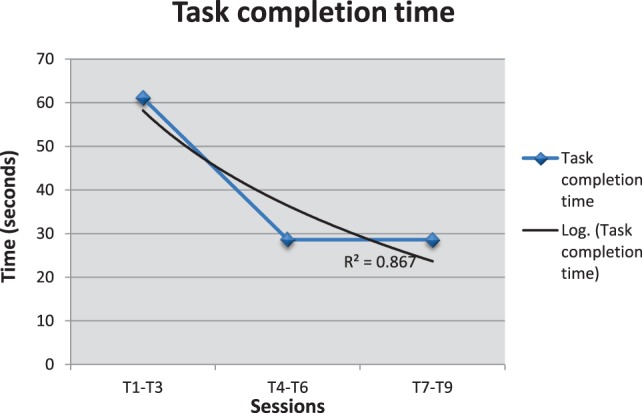
**Evolution of the time (in seconds) to complete the drawing in Imitation game 1**. Evolution of the time occurred to complete the Imitation game 1 for one child across different sessions. Figure shows that the child becomes faster at reproducing the drawing model.

**Figure 8:**
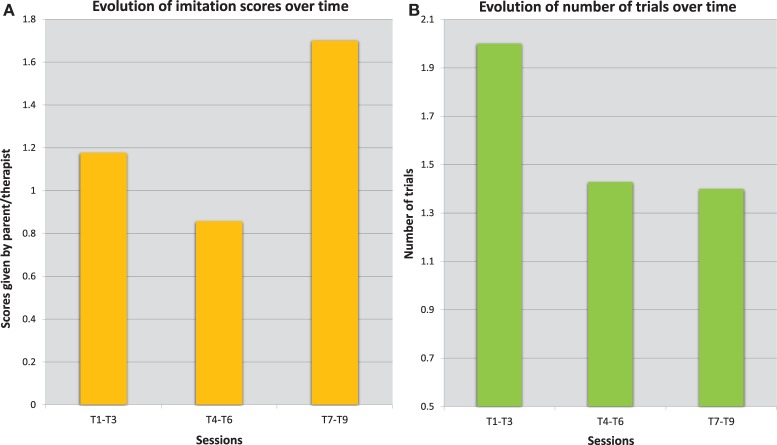
**Evolution of the performances of one child during the Imitation game 1**. The error bars **(A)** describes the variations of the scores given by the therapist at hospital for different sessions. The quality of imitation improved throughout the sessions: the average score (av = 1.7) during the third period (T7–T9) is closer to the maximum score (score 2) and higher than the initial scores for the periods T1–T3 (av = 1.2), and T4–T6 (av = 0.9). The average number of trials required to complete the imitation, shown on the right **(B)**, has decreased as well across different sessions from the first period (T1–T3 with av = 2) to the next periods (T4–T6 and T7-T9 with av = 1.4).

**Figure 9:**
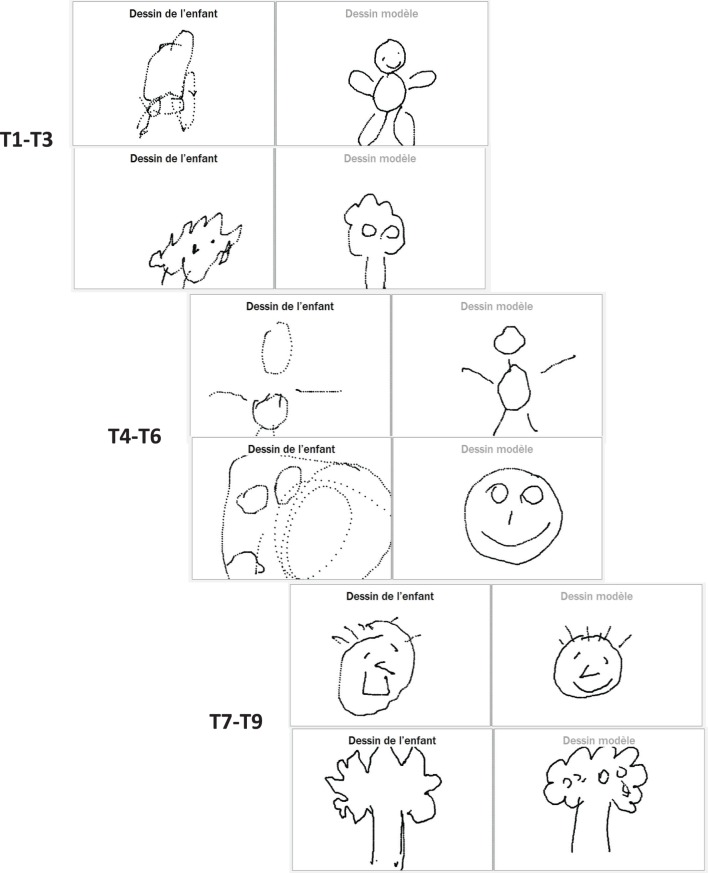
**Evolution of the imitation skills of a child across three periods**. Example of the evolution of the imitation skill for one of the children across different periods.

Figure 7 shows that the child becomes faster at reproducing the drawing model (*R*^2^ = 0.867). In addition, the quality of imitation improved throughout the sessions as shown by the evolution of the imitation scores (given by the therapist/parent) in Figure 8. The quality of the imitation is evaluated by the therapist/parent among three possibilities: correct (score 2), partially correct (score 1), and incorrect (score 0). Figure 8A shows that the average score (av = 1.7) during the third period (T_7_–T_9_) is closer to the maximum score (score 2) and different from the initial scores for the periods T_1_–T_3_ (av = 1.2) and T_4_–T_6_ (av = 0.9). Furthermore, as shown in Figure 8B, the child needed fewer trials to reproduce the therapist/parent’s drawing. As an illustration, Figure 9 represents the evolution of child’s imitation skills in drawing across the three periods.

### Parents Experience and View

**Figure 10:**
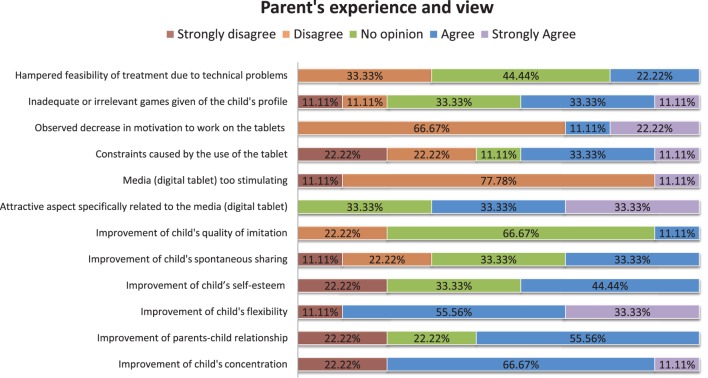
**Results related to the questionnaire proposed to the parents**. Answers given by the parents of the children recruited for the study to the questionnaire containing the questions related to the use of the GOLIAH platform.

At the end of the 3-month open trial, a web questionnaire was sent to the parents of children who participated in the open-trial (10 parents). The questionnaire contained 12 questions with a positive or negative orientation toward the serious game (see details at https://goo.gl/foMpPI). The questions asked about the use of the game (ease of use for parents, chosen media, technical problems, etc.) and the improvement in the child’s skills (concentration, attention, imitation, self-esteem, etc.). The parents had to answer through a Likert scale from 1 to 5 (1 = strongly disagree, 2 = disagree, 3 = no opinion, 4 = agree, 5 = strongly agree). Results are summarized in Figure 10 and show that parents have positively assessed the use of the serious game as a treatment. Sixty-seven percent of interrogated families did not observe a decrease in the child’s motivation to work on tablets; 44% of them were not particularly disturbed by the constraints on daily activities caused by the use of the serious game on tablets, and 33% judged that the feasibility of treatment was not seriously hampered due to technical problems. The media (digital tablet) was not considered as too stimulating by 89% of the families and more than 67% of them thought that there was a specifically attractive aspect related to the media itself. Only one negative point was noted: 44% of the parents found that the games were inadequate given their children’s profile. At the beginning of our pilot study, we were aware of this possible limitation. However, since our focus was to assess the feasibility and usability of the game, older participants were preferred because they could be more willing to collaborate and test the game.

Concerning progress on the children’s skills, it seems that there is not so much progress on Imitation since the majority of the parents (67%) had no specific opinion on this topic. On the contrary, JA (spontaneous sharing) seemed to be slightly ameliorated (33% agreement). Interestingly, some skills that were not directly trained by the games strongly evolved during the course of the 3-month open trial according to parents: child’s self-esteem, child’s concentration, and child’s flexibility. Moreover, the quality of parents–child relationship was qualified as enhanced for 56% of the parents. We could hypothesize that the interactive nature of GOLIAH and its pleasantness for the child had the effect of improving parent–child interaction also in other contexts, which is a generalization effect that often is lacking in treatments for autism.

## Conclusion

In the current paper, we described a gaming platform for home-based intervention in ASD. Within the context of a pilot open trial, we showed the feasibility of the intervention. We found that (1) the gaming platform was useful during both children–therapist interaction at hospital as well as children–parents interaction at home, (2) tailored intervention was compatible with at home use and non-professional therapist/parents, (3) children performed as expected when using the different Imitation and JA games and no game appeared inaccurate, (4) data computed from the platform and clinical annotations produced by parents and therapists allowed session-to-session monitoring and helped therapists to dynamically reconfigure treatment, and (5) subjective views from users (mainly parents here) were overall positive. From the clinical point of view, the most important benefits of this novel method of intervention for children with autism are: (a) the rapid performance amelioration on tasks based on Imitation and JA that are considered pivotal for children with autism; (b) to create a scenario where the spontaneous, and usually lone, activity with video games is easily pushed to become a shared activity; (c) a general amelioration of attention and availability to discuss the results of a performance. Nevertheless, some limitations must be considered. First, the lack of more precise and external evaluation of improvements in Imitation and JA with specific methodology; second, a deeper analysis of the minority of parents who have signaled difficulties in applying GOLIAH is needed to individuate for which child and for which family it could be more indicated; third, in a future study, it will be important to study the gender differences than the current GOLIAH tasks and to evaluate the appropriateness of the GOLIAH tasks also with girls with ASD. Given the promising preliminary results, we are moving now within the context of FP7 MICHELANGELO project to further ascertain the efficacy of the gaming platform in the context of a bigger (*N* = 30) and longer (6 months) clinical trial, including a control group. Besides Imitation and JA, two cognitive skills directly targeted within the gaming platform, we plan to use external primary variables (i.e., Vineland scores and Social Communication Questionnaire) to assess generalization.

## Author Contributions

VB created the entire gaming platform with help from WJ and SH. KM and MW first conceptualized the gaming platform and KM coordinated the work. AN made critical evaluations of the stimuli to be selected from ESDM protocol, recruited and evaluated the children in Pisa, and ran the open trial; A-LJ adapted ESDM stimuli in serious game stimuli; JX recruited the children in Paris; ET recruited and evaluated children in Paris and ran the open trial; MC, DC, and FM provided supervision and reviewed the paper. The MICHELANGELO study group contributed to the overall project and study design, help in managing computational data and engineering issues.

## Conflict of Interest Statement

The authors declare that the research was conducted in the absence of any commercial or financial relationships that could be construed as a potential conflict of interest.

## Supplementary Material

Presentation 1 **This file contains the description of the other nine games of the gaming platform which have not been described in the paper**.

Data Sheet 1 **This file contains the data used for the analysis of the games Bake a recipe and Free Drawing presented in the Results and Discussion section**.

### Availability of Data and Materials

The data used to represent the results of the games Bake a recipe and Free Drawing can be found in the Supplementary material as Data Sheet 1.

## Appendix A

### MICHELANGELO Study Group

Silvio Bonfiglio (FIMI, Italy), Fabio Apicella, Federico Sicca (Fondazione Stella Maris, Italy), Giovanni Pioggia (CNR, Italy), Federico Cruciani, Cristiano Paggetti (I + , Italy), Angele Giuliano, Maryrose Francisa (Accros Limit, Malta), Saptarshi Das (University of Southampton, UK), Leo Galway, Mark Donnelly (University of Ulster, UK).
