# Supplementary material for: GOLIAH: A Gaming Platform for Home-Based Intervention in Autism – Principles and Design
Source: Front Psychiatry. 2016 Apr 28;7:70. doi: 10.3389/fpsyt.2016.00070 (PMC4848303; doi:10.3389/fpsyt.2016.00070)
Supplement: Presentation 1 — This file contains the description of the other nine games of the gaming platform which have not been described in the paper. [file presentation_1.PDF]

Supplementary Materials – File 1

GOLIAH: A Gaming Platform for Home-Based Intervention in Autism – Principles and Design

Valentina Bono, Antonio Narzisi, Anne-Lise Jouen, Elodie Tilmont, Stephane Hommel, Wasifa Jamal, Jean Xavier, Lucia Billeci, Koushik Maharatna, Mike Wald, Mohamed Chetouani, David Cohen, Filippo Muratori and MICHELANGELO Study Group

Correspondence: Valentina Bono: vb2a12@ecs.soton.ac.uk

Imitation game 2 – Imitate step by step drawing

This game is similar to the imitation game 1, described previously, but it has different levels of difficulty:

1) In the *step by step* difficulty, the therapist/parent would draw an object in several steps. For instance, a house could be sketched by drawing, first a square, then adding a triangle on the top, and so on. At every step, the player will copy the therapist/parent's sketch. The therapist/parent may draw more complicated shapes at each step after seeing the imitation ability of the player in the previous drawing.

2) In *end-step by step* and *snapshot at the end*, the therapist/parent first draws all the steps required to complete the drawings. The player will imitate the sketch after the appearance of the sequence of steps (*end-step by step*) or the complete drawing (*snapshot at the end*).

In all the difficulty options mentioned above, the therapist/parent can accept the player's drawing or can encourage him/her to give another try at any intermittent step. When the therapist/parent determines the end of the game, he/she will evaluate the player's imitation ability among the three options: correct, incorrect or partially correct.

Imitation game 3 – Imitate Speech

The aim of this game is to make the player imitate the sound of recognizable single words or phrases. The player has to first hear and then repeat the sound of the words or sentences selected by the therapist/parent from the standardized library. The therapist/parent can select the stimuli among three levels of difficulty: (1) *words* (e.g. “mother”), (2) *simple phrases* with few words (e.g. “I want”) or (3) more complicated *phrases* (e.g. “my mother is at home”), by selecting the appropriate option from a drop-down menu in his/her device. The selected words or phrases will be played on the player’s device who, after each sound, will imitate the word or the phrase. The player’s imitation is recorded automatically and played back to the therapist/parent for the evaluation. The response will be stored in the player’s device to allow the therapist to investigate offline the parent’s evaluation (if any) when the game is played between the child and the parent. As in the previous games, the assessment of the imitation’s quality is performed by the therapist/parent among the three available choices as shown in Figure 1.

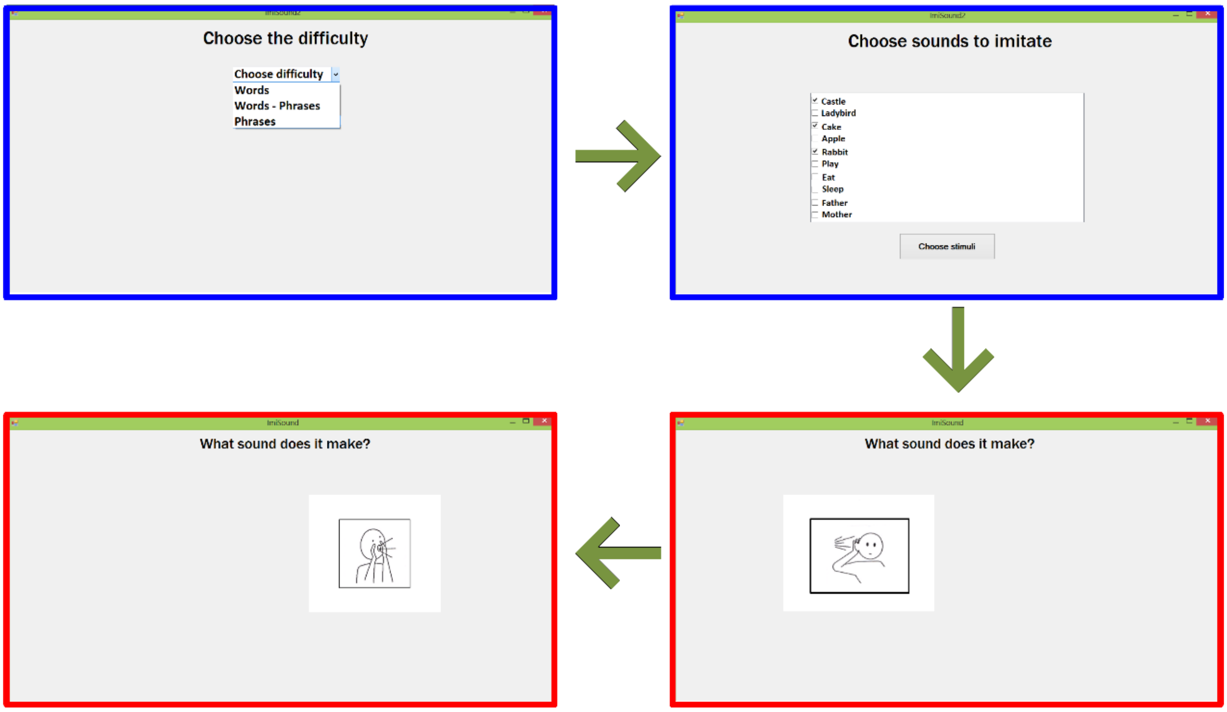

Figure 1 - Flow of the Imitation game 3 – Imitate Speech

The therapist/parent selects the difficulty and the list of words to be imitated (blue windows); the player will listen to the sound and imitate it (red windows).

Imitation game 4 – Imitate Sound

This sound imitation game allows the analysis of the player's skills in the imitation of sounds of (1) *everyday objects* or (2) *animals*. The whole process of this game is shown in Figure 2**Erreur ! Source du renvoi introuvable.**. The therapist/parent first selects the appropriate stimulus by clicking either on the animal icon or the object icon in his/her window. As shown in Figure 3 (the example shows the available options when the therapist/parent clicks on the animal icon), this will open the available options already stored in a pre-defined library on his/her device from which the therapist/parent can select the appropriate stimuli. Currently the two libraries for the *everyday objects* and *animals* include the sounds of train, scooter, donkey, pig, etc. The selected sounds will be reproduced in different ways, according to the selected level of difficulty from a drop-down menu: (1) *human imitation and picture*, where the sounds imitated by a third person will be reproduced together with the picture of the chosen item, (2) *true sound and picture*, in which the picture is associated with the real sound of the stimulus, (3) *true sound*, in which only the sound is presented without showing any picture, and (4) *picture*, where only the picture of the object or animal is shown, without any sound reproduction.

Following the presentation of each selected stimulus, the player will repeat the sound. The recordings acquired on the player's device, will then be played on the therapist/parent's device and the therapist/parent will score the performance following the same procedure of the previous games. As with the Imitation game 3, the responses are recorded in the player's device to allow the therapist to investigate the parent's evaluation offline at a later stage.

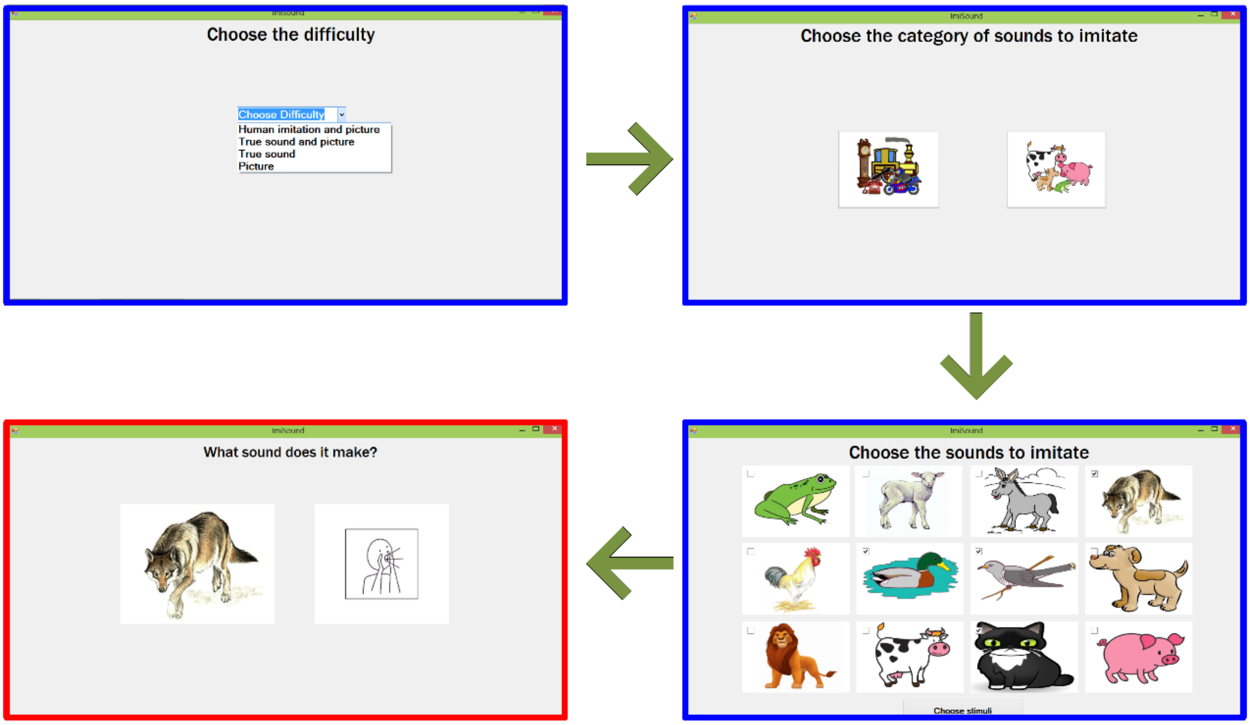

Figure 2 - Flow of the Imitation game 4 – Imitate Sound

The therapist/parent selects the difficulty, the category and the stimuli to imitate (blue windows) and the player will imitate after hearing the sound and/or looking at the picture (red window).

Imitation game 5 – Imitate Actions

The goal of this game is the imitation of actions with an object initiated by the therapist/parent in multiple steps. As shown in Figure 3, the therapist/parent can select the type of imitation between (1) *each step imitation*, where the child has to imitate the action performed by the therapist/parent at each step, and (2) *full action imitation*, in which the child will imitate the actions only after seeing all the steps performed by the therapist/parent. The game involves a minimum of three action steps: the therapist/parent and the player have to open a box by moving a lid to a dedicated space on their respective panels, put one or more balls into the box and close the box, and put the lid back on the top of the container. The therapist/parent can select among three different levels of difficulty, depending on the number of balls to move during the actions: (1) *one*, (2) *two* or (3) *three balls* can be put into the box.

At the end of each step or at the end of the game, according to the selected task (*each step* or *full action imitation*), the actions imitated by the child, will be shown in the therapist/parent's device on the right panel.

The extent of the imitation will be evaluated by the therapist/parent among the three options: correct, incorrect or partially correct imitation. Like the other games with a manual assessment, the final image showing both therapist/parent and player's actions is saved for further checking.

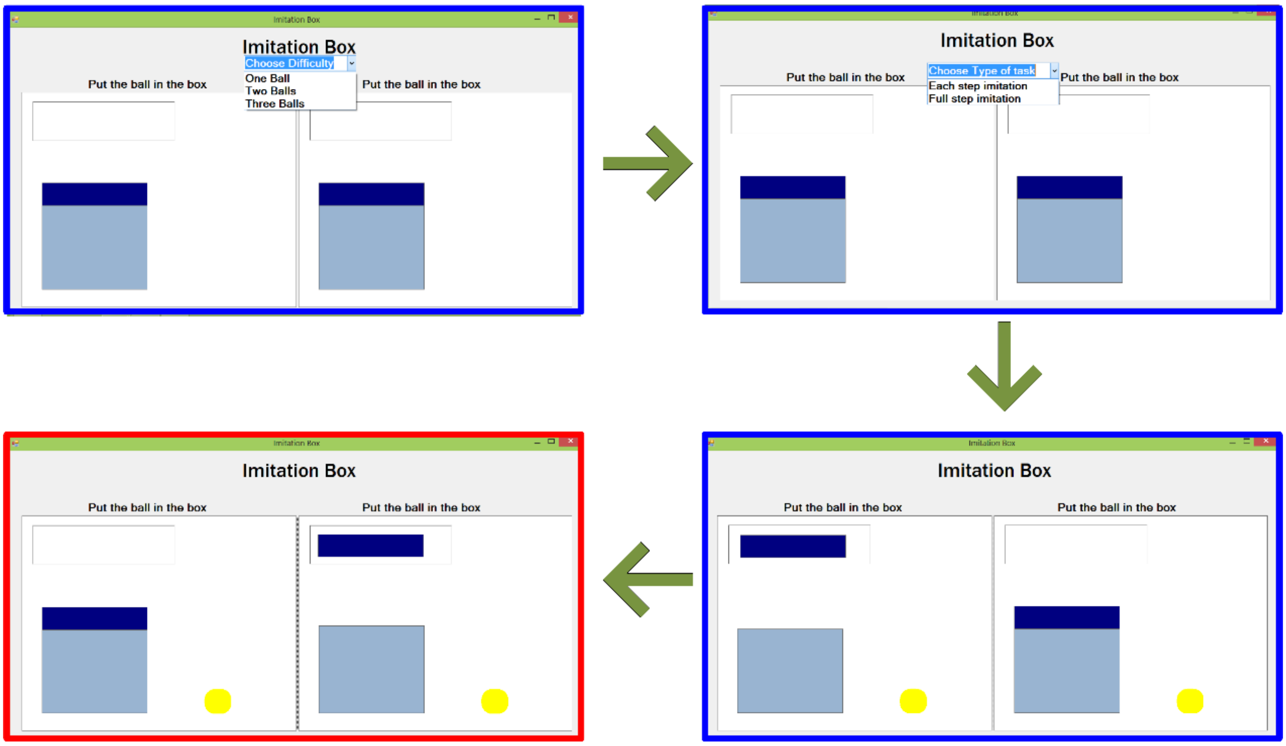

Figure 3 - Flow of the Imitation game 5 – Imitate Actions

After choosing the difficulty level and the type of imitation, the therapist/parent (blue windows) will move the lid of the box. This action will be shown on the right panel of the player's window (red window) who will then imitate it on the left panel.

Imitation game 6 – Imitate Actions and Build

The purpose of this game is to imitate and build objects by moving the cubes, as shown in Figure 4. The player's imitation skills can be analysed by following two different procedures: (1) the player can emulate the action at each step, in case of *each step imitation*, or (2) he/she can emulate the actions after the therapist/parent has finished moving all the cubes, in case of *full action imitation*. Three or more cubes are used to build the objects, according to the selected difficulty: (1) a bridge could be built by using *3 cubes*, (2) a tower by using *4 cubes* or (3) *10 cubes* will be used to build a pyramid. According to the selected task, the single step or all the steps will be displayed in the player's device, who will imitate the actions performed by the therapist/parent. As in the previous game, the therapist/parent will evaluate the player's performance after the imitated actions are displayed on his/her device. The final image showing both the therapist/parent's and the player's actions will be saved for further checking.

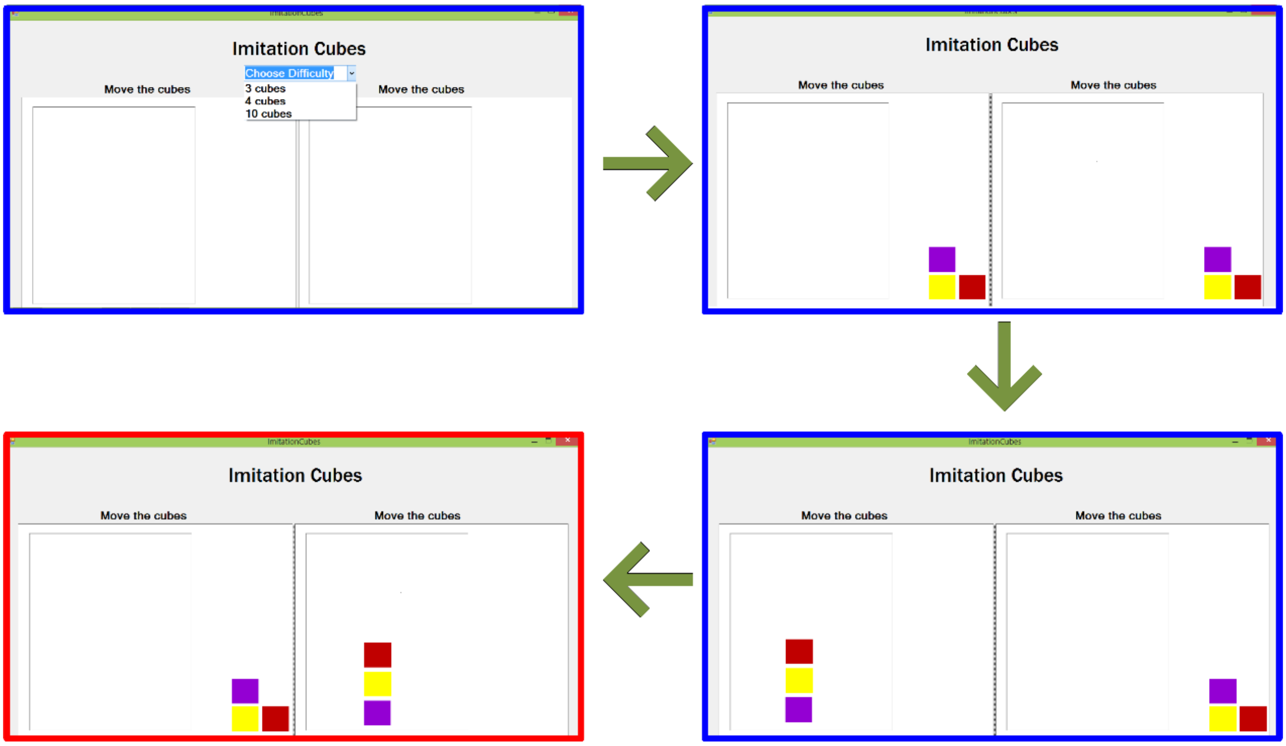

Figure 4 - Flow of the Imitation game 6 – Imitate Actions and Build

The therapist/parent (blue windows), after selecting the number of cubes to utilize, will build an object on his/her left panel. This action will show on the right panel of the player's device (red window) who will then imitate it on the left panel.

Imitation game 7 – Guess the musical instrument

The purpose of the game is the imitation of a sequence of musical sounds selected by the therapist/parent. The therapist/parent can select the number of musical instruments to show, according to the chosen level of difficulty, (1) *3 musical instruments* or (2) *6 musical instruments*. According to the starting skills of the player, the therapist/parent may select a sequence of one to six musical instruments by clicking on the icons as shown in Figure 5. The selected music is then played on the player's device: the corresponding instruments need to be identified and clicked by the player on his/her window. The evaluation of the player's skills is automated: each correct answer will produce an event labelled with a unit score. In case the player has difficulties in guessing the instrument, the sound could be played back once again by clicking the Play button. The number of errors and the number of replays would be saved, allowing further analysis to track the player's improvements over time.

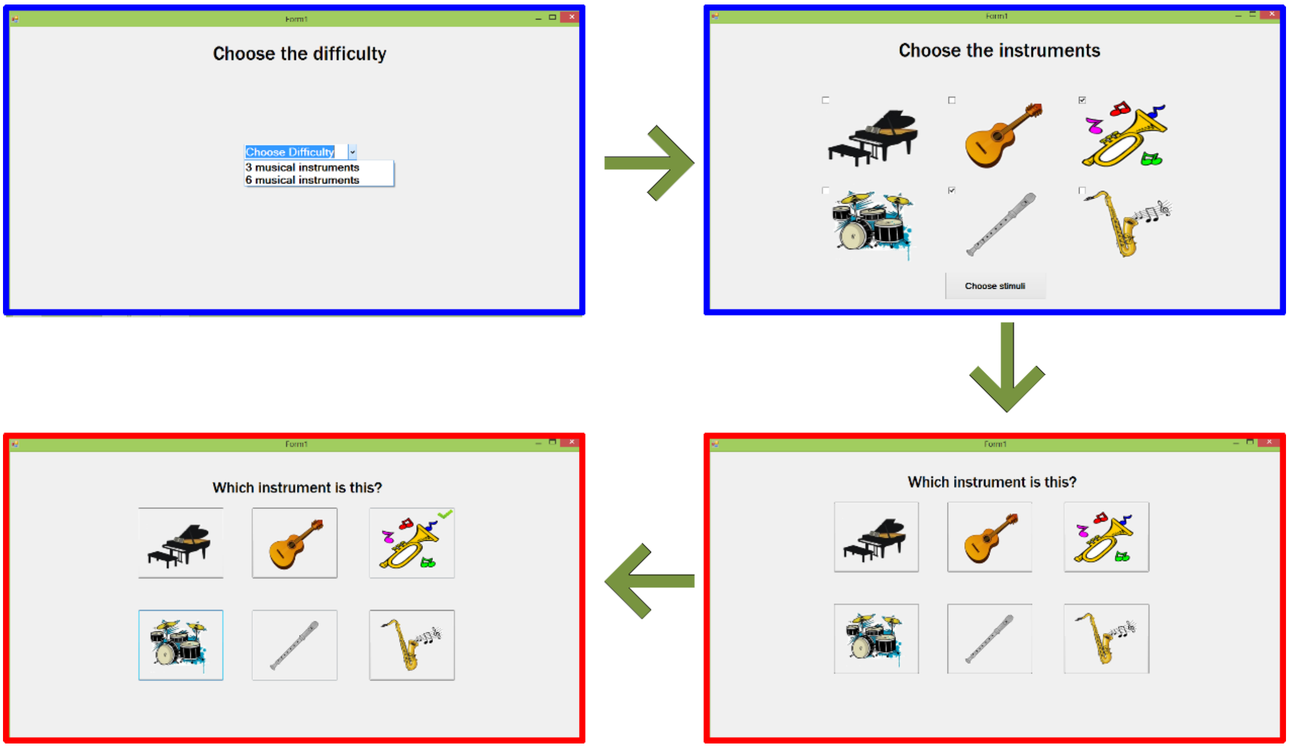

Figure 5 - Flow of the Imitation game 7 – Guess the musical instrument

The therapist/parent (blue windows) selects the number of instruments to be showed on the player's device and selects the instruments to be played on the player's device (red windows). After listening to the sequence of instruments, the player will click on the corresponding musical instruments.

Joint attention game 1 – Follow pointing (both audio and visual)

This game is based on different JA stimuli from the ESDM such as looking at a picture indicated verbally, pointed with a finger or with a gaze shift. As shown in Figure 6, the game consists of a library of pre-recorded videos in which a person indicates an object following different procedures, according to the related difficulty levels: (1) *point only*, (2) *speak and look*, (3) *speak only*, (4) *point and look*, (5) *look only* or (6) look but in the meanwhile point at the opposite direction (*inconsistent condition*). The therapist/parent selects the following criteria: the indication procedure, the number of objects that will be indicated, as well as the type of objects. In fact, the therapist/parent can adopt to play with two, four, six or eight objects which can be squares, cartoons, superheroes, food, cars, etc. As soon as the options described above are selected, the therapist/parent will select the first item and, consequently, an appropriate video from the pre-recorded library showing a person indicating the selected object will appear in the player's device. This approach mimics human-human interaction to some extent giving the impression to the player that he/she is interacting with a person and not with a mere machine. After watching this video, the player will then have to click on the corresponding item. In case of failure, e.g. the player clicking on the wrong item, the video is played again to help him/her to respond correctly. An automated event for each correct or wrong answer is created, respectively with a positive or negative score.

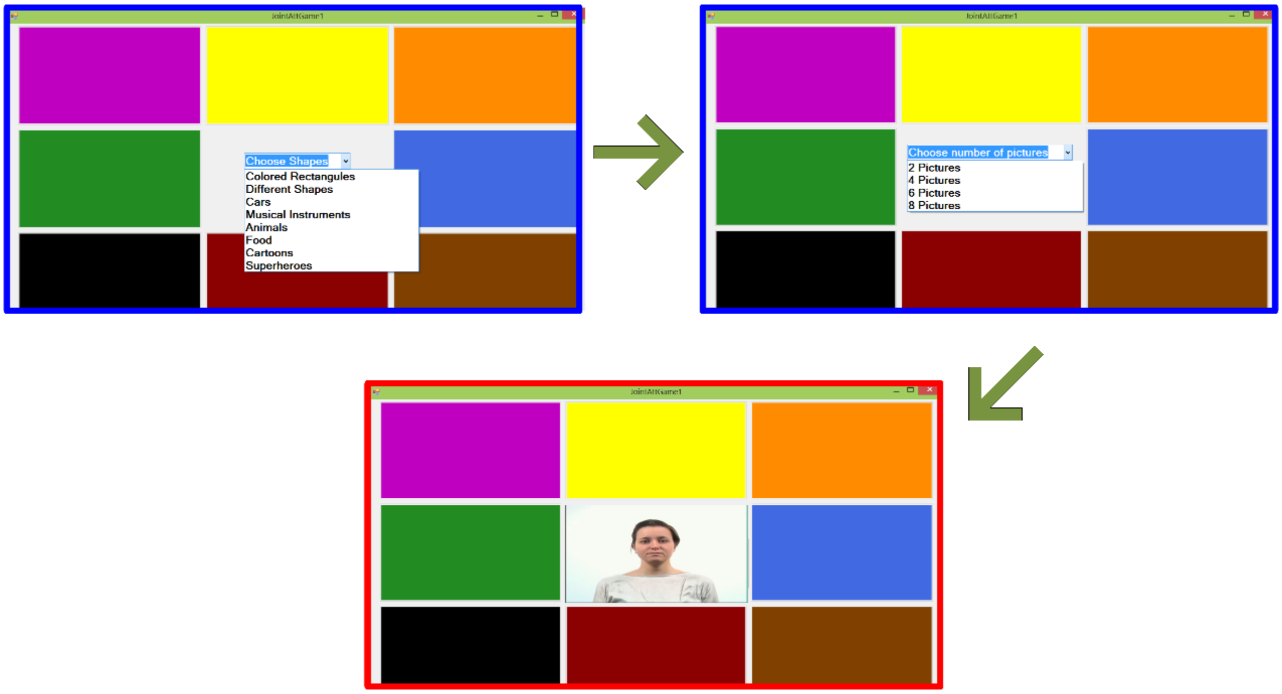

Figure 6 - Flow of the Joint Attention game 1 – Follow pointing (both audio and visual)

The therapist/parent (blue windows) will select the shape of the object to be pointed on the video and the difficulty level. The player's will watch the video and click on the pointed location of the object.

Joint attention game 2 – Cooperative drawing

The aim of the cooperative drawing game is the creation of a figure or shape by cooperatively connecting the dots on its edges, as shown in Figure 7. At the beginning of the game, the therapist/parent selects the figure from a library which includes two difficulty levels, (1) *easy* and (2) *hard*. The figure can be (1) a *letter of the alphabet*, (2) an *object* (like a tree), (3) a *geometric shape* or (4) a *number*. In both the therapist/parent and player's devices, the window is divided into two panels (similar to the drawing imitation games): on the right side the final figure is shown and on the left side the dots and lines will appear in real time each time a player clicks and/or connects two dots. The therapist/parent starts by clicking a dot, after which the player and the therapist/parent alternatively put a new dot next to the previous one in order to connect the line and complete the final figure shown on the right. An event with a positive or negative score will be produced each time the child attempts to put a new dot and, therefore, connect a new line. The player's skills evaluation will be made at the end of the game with a final score.

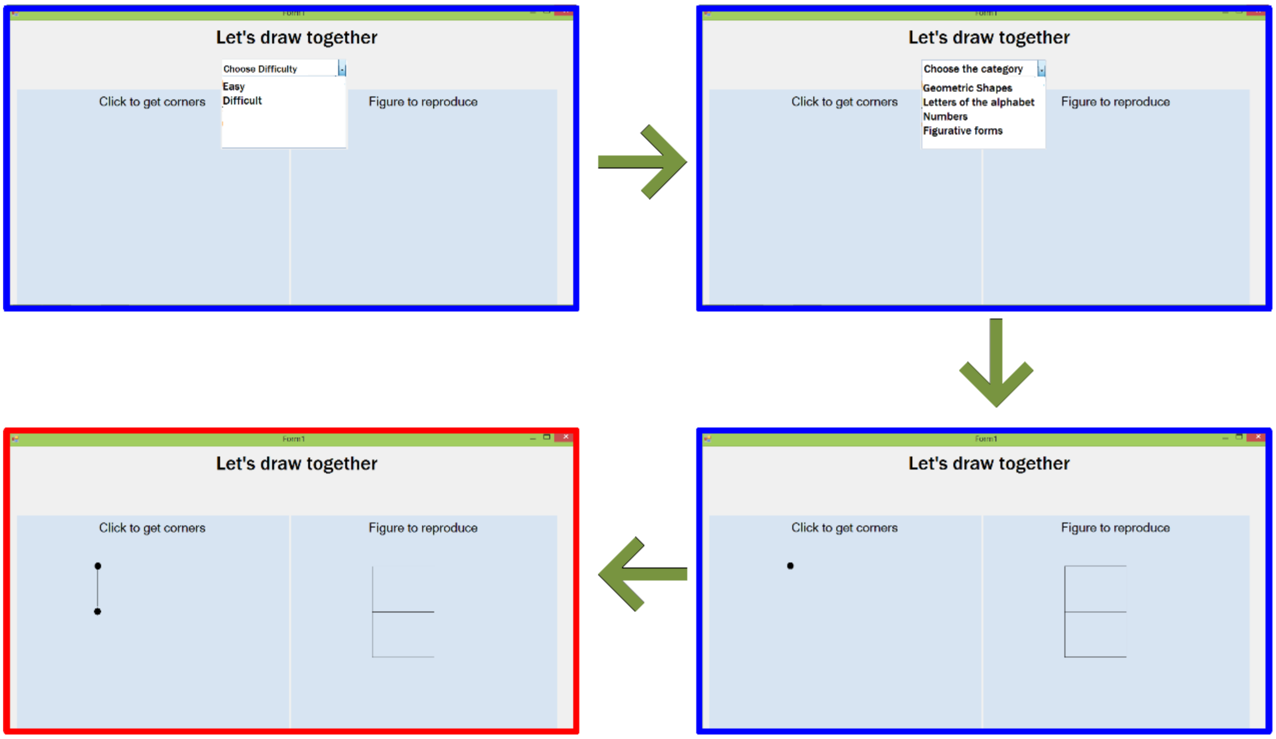

Figure 7 - Flow of the Joint Attention game 2 – Cooperative drawing-connect dots

The therapist/parent (blue windows), after choosing the number to cooperatively draw, will click on an edge of the number. The player (red window) will click on the closest edge to draw the line. The game will continue until the number 8 will be completed.

Joint attention game 4 – Receptive communication

This game is based on various JA ESDM stimuli, including the identification and the viewing of a named picture. As shown in Figure 8, the difficulty options of this game are based on the number of pictures that will be shown on the player's device: the player has to identify and click the object described by the therapist/parent that will be illustrated among (1) *3 objects*, (2) *5 object* and (3) *8 objects*. The therapist/parent selects the item from the library which consists of five categories of pictures: (1) *animals*, (2) *furniture*, (3) *vehicles*, (4) *fruits* and (5) *vegetables*. Once the therapist/parent picks all the pictures to be described; the player will see those pictures on his/her device and will have to identify and click on them. For each item, the event related to the correct or incorrect answer is produced with the related positive or negative score.

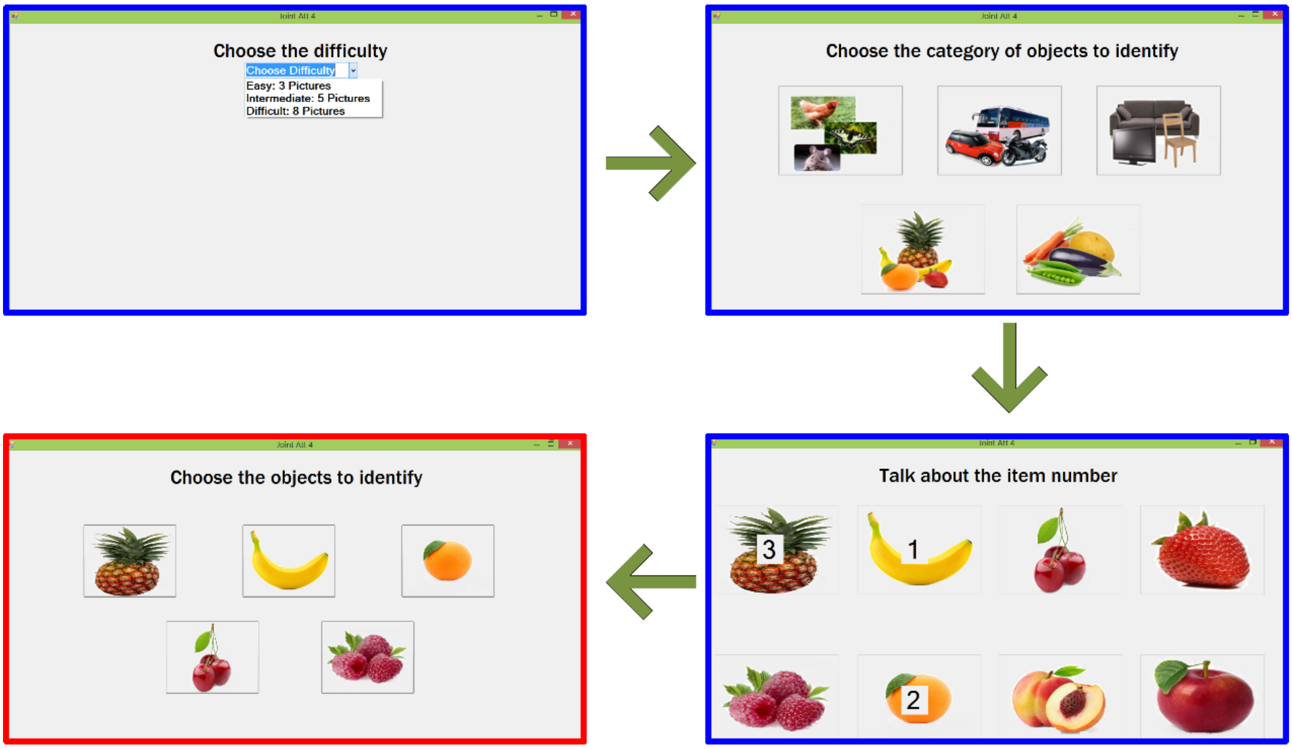

Figure 8 - Flow of the Joint Attention game 4 – Receptive communication

The therapist/parent (blue window) selects to show on the player's device 5 picture (difficulty) and to indicate 3 fruits (category). The player will have to identify and click on the three fruits indicated and explained by the therapist/parent. After dragging all the ingredients, the player's will click on the recipe cooked.
